# Reproducibility of Acute Changes in Range of Motion Following Short-Term Static Stretching of the Hamstrings and Gastrocnemius

**DOI:** 10.3390/jfmk11030373

**Published:** 2026-09-18

**Authors:** Uršula Prelesnik, Žiga Kozinc

**Affiliations:** 1 Faculty of Health Sciences, University of Primorska, SI-6310 Izola, Slovenia; 2 Ludwig Boltzmann Institute for Rehabilitation Research, A-1140 Vienna, Austria

**Keywords:** reliability, range of motion, static stretching, responders, non-responders, change-score reproducibility

## Abstract

**Background and Objectives:** Acute responses to exercise interventions may vary within individuals across repeated exposures, potentially limiting the interpretation of individuals as “responders” or “non-responders”. This study investigated whether acute changes in range of motion following short-term static stretching are reproducible within individuals. **Methods:** Twenty-six healthy young adults completed two identical sessions separated by one week. Flexibility was assessed before and after static stretching using the Sit-and-Reach Test (SRT) and ankle dorsiflexion range of motion (ADF ROM). The intervention consisted of three 30 s static stretches for the hamstrings and gastrocnemius. **Results:** Baseline measurements showed excellent reproducibility (SRT: ICC = 0.98; ADF ROM: ICC = 0.95). SRT performance and ADF ROM increased significantly after stretching (*p* < 0.001), with no evidence of a systematic difference in the mean response between visits (time × visit interactions: *p* > 0.05). Reproducibility of individual pre-to-post change scores was moderate by point estimate but imprecisely estimated (SRT: ICC = 0.74; 95% CI: 0.50–0.88; ADF ROM: ICC = 0.57; 95% CI: 0.25–0.78). **Conclusions:** Individual change scores showed limited reproducibility. The design cannot separate measurement error, normal day-to-day variation, and variability specifically attributable to the stretching response. Classifying individuals as “responders” or “non-responders” based on a single acute stretching session should therefore be approached with caution.

## 1. Introduction

In recent years, there has been extensive discussion in sports science regarding “responders” and “non-responders” to a specific exercise intervention. Following the intervention, authors often categorize the participants based on their results into those who responded to the exercise intervention or therapy (“responders”) and those who did not (“non-responders”) [1]. A common problem is that differences in how individuals change following an intervention are often assumed to reflect true differences in trainability. This can lead to participants being classified as “responders” and “non-responders,” even when the observed variability may partly reflect measurement error or normal within-individual fluctuations [2,3]. Inter-individual differences in trainability refer to differences in outcome measures between individuals attributable to the intervention itself (i.e., differences caused by the intervention per se) and should be distinguished from variability caused by random measurement error and intraindividual/within-subject variability [1,4]. Numerous articles [3,5,6,7,8] argue that authors often lack adequate statistical approaches when interpreting final results regarding variability in response between individuals and fail to consider certain important statistical measures when determining individual responses (e.g., measurement error, the smallest worthwhile change/minimal clinically important difference).

The typical error (TE) reflects random measurement error arising from both the measurement process and normal biological variation [1]. When comparing pre- and post-intervention values, individual changes that do not exceed the TE may simply reflect measurement “noise” and cannot necessarily be interpreted as an improvement in the outcome variable—therefore, all changes that do not exceed the TE could, in fact, be classified as “non-response” [9,10]. However, when participants’ responses exceed the TE, it could be argued that two responses differing by less than the value of the TE are not necessarily truly different from one another [11]. Many studies have failed to take the TE into account, leading to an overestimation of the response rate (i.e., a greater number of individuals being classified as “responders”) compared with what would be observed if authors used a stricter threshold for defining “responders” that accounted for both the TE and the smallest worthwhile change/minimal clinically important difference [1]. Considering the minimal clinically important difference is important because a change exceeding the TE does not necessarily imply that this change is clinically relevant (i.e., an actual improvement in disease status or reduction in disease risk) or meaningful from the perspective of sports performance [11]. The minimal clinically important difference is specifically determined for each variable based on available evidence; when such data are unavailable, the use of the smallest worthwhile change is recommended [1,12]. Bonafiglia et al. [1] systematically reviewed methods used to assess interindividual differences in trainability and individual responses to exercise. They concluded that many studies may have overinterpreted response variability as evidence of “responders” and “non-responders” because appropriate statistical approaches were not adequately applied. As a result, the extent to which true interindividual variability in trainability exists remains uncertain, with the available evidence being mixed. Similar conclusions were reached by Xiao and Ren [13] in a systematic review of aerobic exercise responses.

Another limitation of classifying individuals as “responders” and “non-responders” is that responsiveness may depend on the outcome assessed. For example, an individual may improve in VO2max but not in body fat percentage [14], and could therefore be classified differently depending on the outcome variable [11]. Responsiveness may also depend on the intervention dose, as individuals who do not respond to a lower dose may respond to a higher one [15]. Thus, if such classifications are used, they should refer to a specific outcome and intervention protocol rather than to general responsiveness to exercise [16]. Responses may also vary when the same intervention is repeated within the same individual [17]. This intraindividual response variability refers to variation in the magnitude of pre-to-post-intervention changes across repeated exposures to the same intervention, beyond random measurement error [7]. It may arise from physiological fluctuations associated with behavioural and environmental factors such as sleep, nutrition, and physical activity [4]. Although relatively understudied, intraindividual variability may substantially contribute to the apparent differences in responsiveness between individuals [7,18], making it difficult to determine how much reported interindividual variability reflects true differences in trainability [1]. Importantly, a control group alone cannot distinguish intraindividual from interindividual variability; the same intervention must be repeated within the same participants [19]. This concept is also described as the repeatability of the response or reliability of the intervention effect [7,18].

Studies of cardiorespiratory fitness and skeletal muscle adaptations have shown that individual responses may not be reproducible when an identical exercise intervention is repeated [17,18,20,21]. This further challenges the classifications based on a single intervention, as an individual classified as a “non-responder” initially may show a positive response upon repeated exposure because of natural within-individual fluctuations [13]. The present study examined this issue using acute changes in range of motion following short-term static stretching of the hamstrings and gastrocnemius. Although a single bout of stretching is well established to acutely increase flexibility [22,23], the reproducibility of individual pre-to-post change scores remains largely unexplored.

Acute increases in range of motion following static stretching are generally attributed to changes in stretch tolerance and musculotendinous mechanical properties [24,25]. Because these perceptual and mechanical determinants can fluctuate across occasions, the magnitude of the acute response may also vary within the same individual. However, evidence on the repeatability of acute stretching responses remains limited. Bryant et al. [26] reported stable mean hamstring-stretching responses across repeated visits, but reproducibility of individual pre-to-post change scores was not the primary focus. It therefore remains unclear whether an individual’s acute response is reproduced when the same stretching stimulus is repeated.

In the present context, we distinguish test–retest reliability of the measurement itself (i.e., stability of baseline values across visits) from reproducibility of the intervention response, which is operationalized as the agreement of pre-to-post change scores when the same intervention is repeated in the same individuals.

The purpose of this study was therefore to determine whether individual pre-to-post change scores following static stretching of the hamstrings and gastrocnemius are consistent across two identical interventions performed one week apart, and to examine the implications for classifying individuals as “responders” and “non-responders” (or as “high responders” and “low responders”) to a given intervention. We hypothesized that static stretching would significantly increase SRT performance and ADF ROM, that the mean pre-to-post changes would be comparable between visits, and that individual pre-to-post change scores would show poor reproducibility.

## 2. Materials and Methods

### 2.1. Participants

Participants were recruited through direct verbal invitations, social media posts, and email invitations distributed to their student or institutional email addresses by the faculty’s administration office. A total of 30 individuals expressed interest in participating. After applying the inclusion and exclusion criteria, 26 participants were enrolled in the study (11 males and 15 females), aged 20–28 years (average age 23.9 ± 2.1 years). Their average height and weight were 171.4 ± 9.2 cm and 66.5 ± 12.9 kg, respectively. Twenty-three participants had a dominant right leg and three had a dominant left leg.

The inclusion criteria were healthy individuals aged between 20 and 30 years, with no history of lower-limb injury or disease. The exclusion criteria were body mass index (BMI) ≥ 30 kg/m^2^, generalized joint hypermobility according to the Beighton score, and/or knee joint hypermobility exceeding 10°. No eligibility restrictions were imposed based on sport participation or previous flexibility training. Participation in the study was voluntary, and participants were free to withdraw at any time. Prior to participation, all individuals were informed about the purpose of the study and the study procedures. No a priori sample-size calculation specifically targeting the precision of the reliability/reproducibility estimates was performed.

### 2.2. Study Design

A repeated measures study design was used, consisting of two identical short-term interventions conducted on the same participants, with measurements performed immediately before and after each intervention. The measurements yielded quantitative data, which were subsequently analyzed statistically. The study was carried out at the Livade 1.0 facility in Izola, Slovenia, at the Faculty of Health Sciences, University of Primorska. All procedures were conducted under the ethical approval of the University of Primorska Human Research Ethics Committee (KER UP; No. 4264-19-6/23; 16 January 2023), granted under the project “Health of the Working Population”.

Participants attended the intervention session (with measurements performed immediately before and after) on two occasions, separated by seven days to minimize carryover from the acute stretching session and conducted at the same time of day. The intervention protocol and all measurements were identical across both sessions. Participants were instructed to avoid lower-limb resistance training for 48 h and any high-intensity physical activity for 24 h prior to each session. Otherwise, they were asked to maintain their usual physical activity levels, dietary habits, and sleep routines. The study was conducted from early November to early December 2025.

The first visit lasted approximately 40 min. Upon arrival, participants changed into comfortable sports clothing that allowed unrestricted lower-limb movement and unobstructed observation of the knees and ankles; shoes were removed for the range-of-motion assessments. Eligibility was verified according to the inclusion and exclusion criteria. The study procedures were then explained in detail, and participants provided demographic information, including age, height, body mass, and dominant leg. In cases where participants were unsure of their dominant leg, dominance was determined using the question: “If you had to hit a target with a ball, which leg would you use to kick the ball?” [27]. A standardized warm-up was performed, consisting of four minutes of step-ups onto a bench at a moderate tempo. Following the warm-up, SRT performance and ADF ROM of the dominant leg were assessed. After the baseline measurements, static stretching of both hamstrings and both gastrocnemius muscles was performed. Following completion of the stretching, all measurements performed prior to it were repeated.

No separate familiarization session was conducted before Visit 1. Participants were familiarized with each procedure during Visit 1 through standardized instructions and, where applicable, practice attempts before the recorded trials.

At Visit 2, participants completed the same standardized warm-up, baseline SRT and ADF ROM assessments, stretching protocol, and post-stretching assessments, using the same procedures, order, and instructions as at Visit 1.

### 2.3. Measurement Procedures

**Figure 1:**
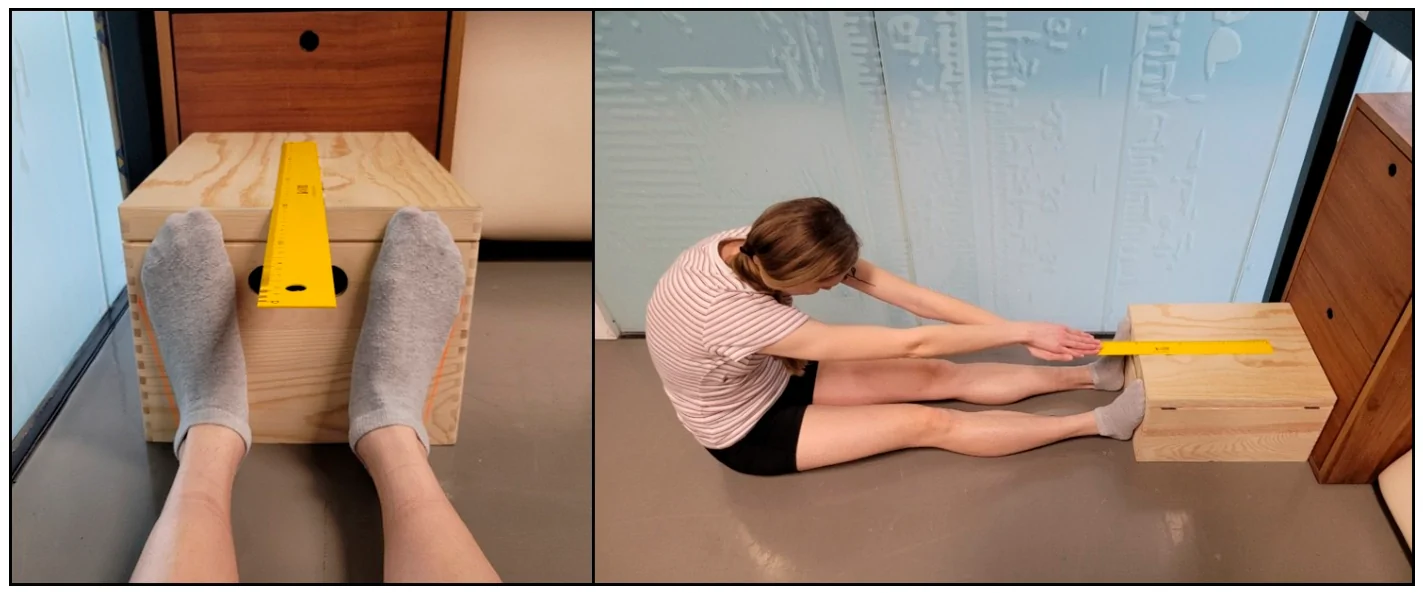
Foot positioning on the SRT device and performance of the SRT.

Baseline measurements consisted of SRT performance and ADF ROM. SRT performance was used as a field-based flexibility outcome and was not interpreted as an isolated measure of hip flexion ROM, because the test is also influenced by pelvic, spinal, and anthropometric factors. SRT performance was assessed first using a rigid custom-made wooden box (40 × 30 × 23 cm) with a fixed 50 cm metal ruler attached to the top surface. The apparatus served as a standardized positional reference for direct measurement of forward-reach distance and was braced against a wall or cabinet to prevent movement during testing. The SRT procedure followed the standardized field-test principles described in the existing reliability literature [28]. Participants were instructed to sit on the floor with knees fully extended, hips aligned with the centre of the box, and feet placed on the box, parallel to marked lines (Figure 1). When sitting upright, participants should still have been able to see the lines, ensuring that the feet did not obscure them. They were then instructed to place one hand on top of the other, interlocking their fingers, and to slowly reach forward along the ruler as far as possible (Figure 1). They were further instructed to hold the final position for approximately 2 s to allow accurate measurement of the reached distance. Forward-reach distance was recorded in centimetres, with values beyond the edge of the box recorded as positive and values short of the box recorded as negative. It was emphasized that the knees had to remain fully extended throughout the movement and that both hands had to move symmetrically to avoid asymmetrical reaching. The movement was continuously monitored to prevent ballistic execution, and to ensure a controlled final position with a 2 s hold. This duration was chosen to allow sufficient time for measurement while minimizing the time spent in a stretched position, thereby reducing any acute stretching effect induced by the test itself. The measurement was repeated three times, and the average value was used for further analysis.

**Figure 2:**
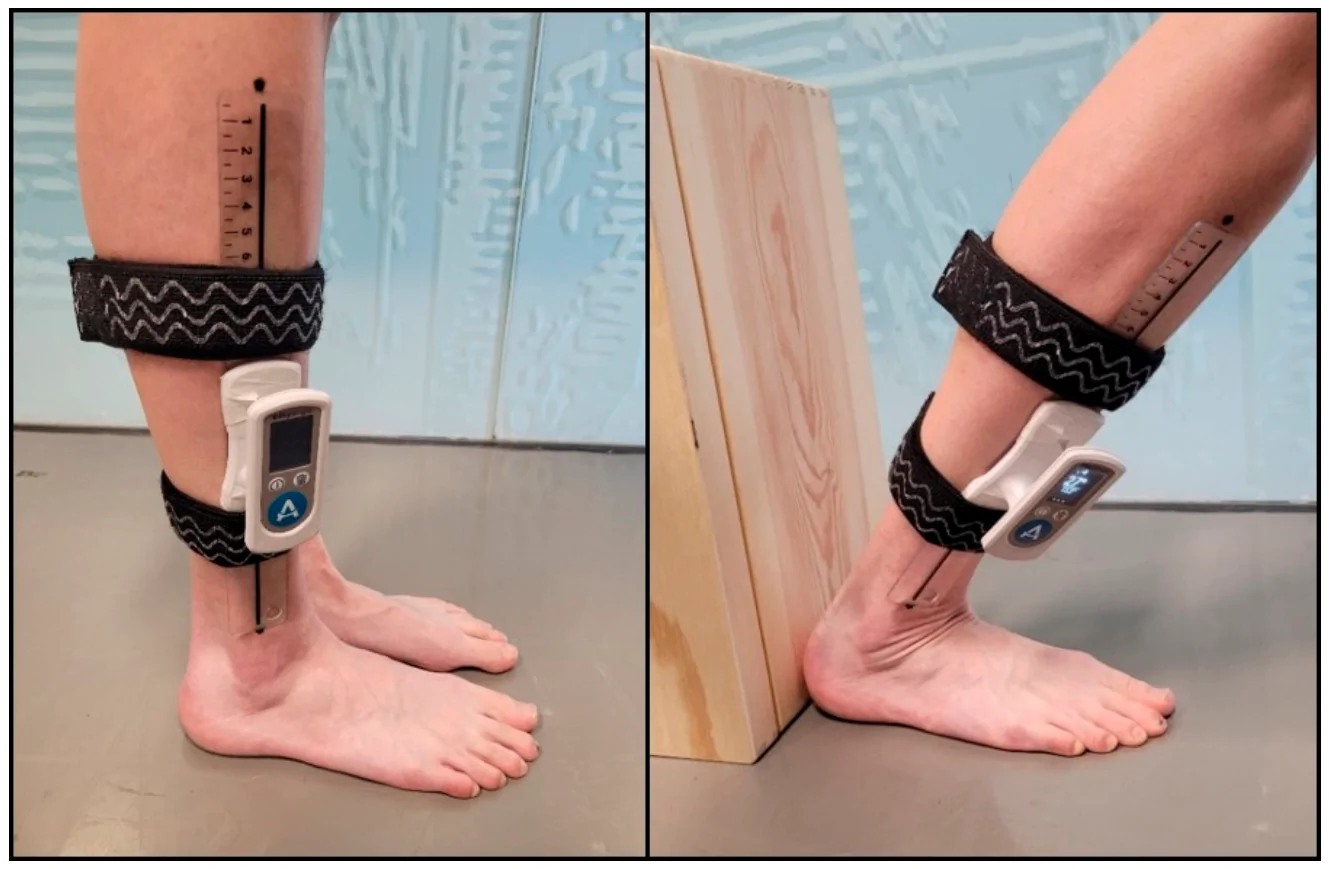
Positioning of the goniometer and measurement of the ADF ROM.

The measurement of ankle dorsiflexion range of motion (ADF ROM) of the dominant leg in a split stance was performed next. A digital goniometer (EasyAngle^®^; Meloq AB, Stockholm, Sweden) was used for this purpose. Prior to the positioning of the goniometer on the lateral aspect of the shank, anatomical landmarks were marked to ensure consistent placement. First, the width of the knee just below the inferior border of the patella was measured using a ruler, and the midpoint was marked with a pen. Then, a point was marked 5 cm proximal to the inferior border of the lateral malleolus, bisecting it. The goniometer was then attached to the participant’s leg using two straps, ensuring that the distal arm was aligned with this distal reference point. The proximal arm was aligned toward the midpoint of the knee using a ruler, and a corresponding mark was made at the proximal end of the arm. The straps were tightened, and the goniometer was carefully aligned with the marked reference points (Figure 2). The participant then stood in front of a table and stepped backward to a self-selected distance, which could be adjusted after a few practice attempts of stepping forward. The feet were placed together and aligned. A box was positioned behind the participant, ensuring that the heels and calves were in contact with it. The examiner stabilized the box with one hand and initiated the measurement on the goniometer with the other hand. The participant stepped forward with the non-dominant leg, grasped the table for support, and leaned forward until a maximal stretch of the gastrocnemius of the dominant leg was achieved with the knee fully extended, without heel lift (Figure 2). Participants were instructed to lean forward without lateral deviation. The knee of the stretched leg had to remain fully extended throughout the movement, and the foot had to remain aligned (no internal or external rotation), with the heel in contact with the ground at all times. Particular attention was paid to heel lift: some participants could perceive the onset of heel elevation themselves (or did not exhibit heel lift at all), while others required verbal feedback from the examiner. Once the final range of motion was reached, the measurement was stopped, and the value in degrees was recorded. The measurement was repeated three times, and the mean value was used for further analysis.

### 2.4. Static Stretching Interventions

Following the baseline measurements, static stretching of both hamstrings and both gastrocnemius muscles was performed. For each stretch, the limb was moved to the participant-reported pain threshold, defined as the point at which the stretch sensation first became painful. The 30 s hold was initiated at this point. The hamstrings were passively stretched by the examiner, whereas the gastrocnemius was actively stretched by the participant. Each muscle was stretched three times for 30 s. If the perceived tension decreased during the hold, the range of motion was gradually increased by the examiner (for hamstrings) or by the participant (for gastrocnemius) until the same pain-threshold criterion was re-established. The same standardized verbal instructions were used at both visits.

Three 30 s repetitions were selected as a conventional short-term static-stretching dose that is sufficient to produce acute increases in range of motion while keeping the intervention brief [22,23,24]. The purpose was to apply the same standardized acute stimulus on two occasions and quantify the reproducibility of the resulting pre-to-post change.

**Figure 3:**
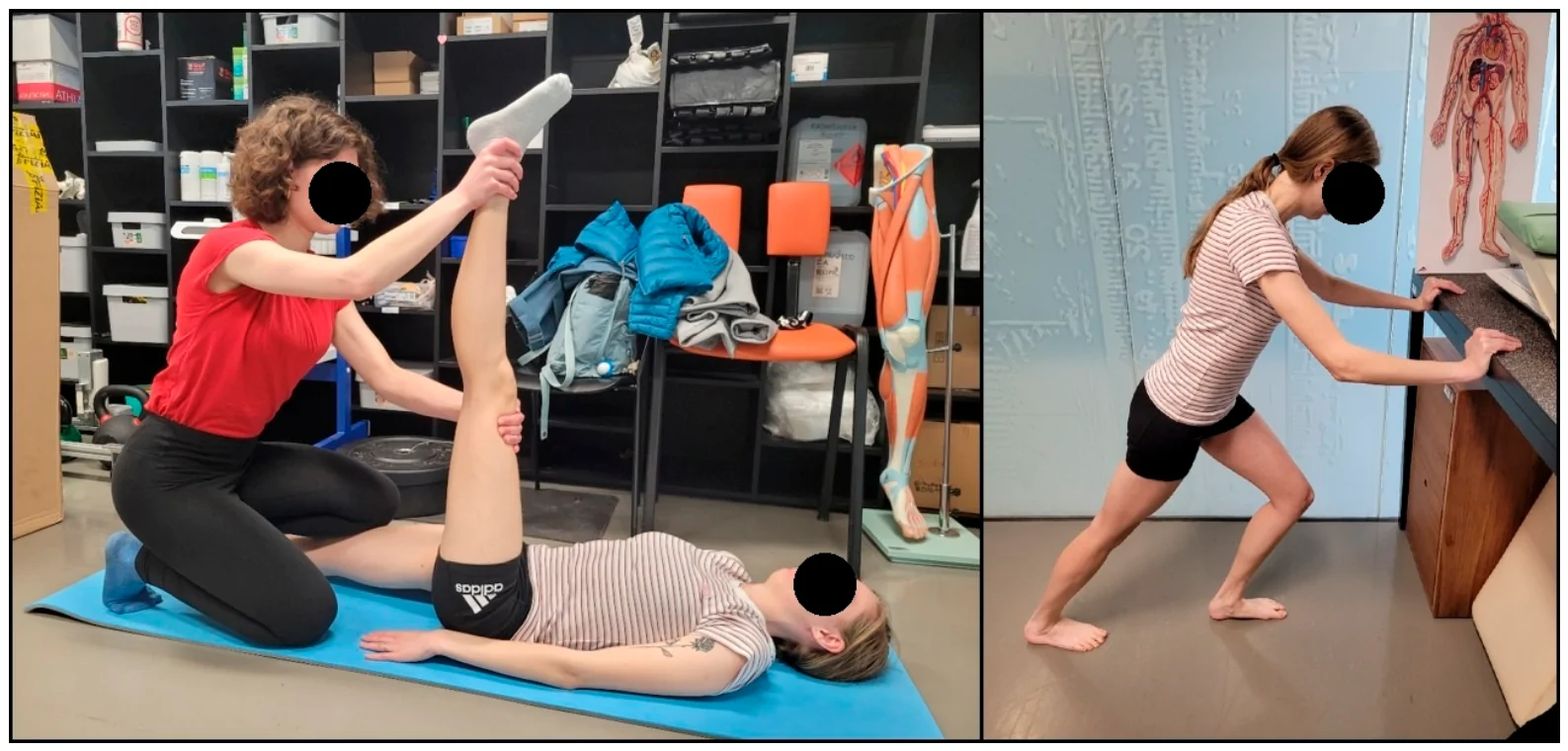
Stretching of the hamstrings (**left**) and the gastrocnemius (**right**).

For hamstring stretching (Figure 3, left), the participant lay in a supine position on a mat and was instructed to relax. The dominant leg was lifted with the knee fully extended, while the contralateral leg was stabilized by the examiner’s knee. The limb was gradually elevated until the participant indicated the point of pain threshold, at which point the 30 s stretch duration was initiated. Participants were reminded during the stretch to report any need for further increase in range. After 30 s, the contralateral leg was stretched using the same procedure.

The participant then stood up and stretched the gastrocnemius muscle of the dominant leg for 30 s (Figure 3, right). The stretching position was identical to that used during the measurement protocol: the participant stood at a self-selected distance from the table, stepped forward with the non-dominant leg, grasped the table for support, and leaned forward until a stretch of the gastrocnemius of the dominant leg was achieved at the point of pain threshold. During the stretch, heel lift was permitted to facilitate a greater range of motion. Particular attention was paid to maintaining full knee extension and proper foot alignment of the stretched leg throughout the entire stretch. Participants were reminded to increase the stretch if necessary. The same procedure was then repeated for the contralateral gastrocnemius muscle. The participant subsequently returned to the supine position, and the sequence of the hamstrings and gastrocnemius stretching was repeated twice more.

### 2.5. Statistical Analysis

Statistical analyses were performed using IBM SPSS Statistics (Version 26.0). Normality of all variables was first assessed using the Shapiro–Wilk test. As no deviations from normality were observed, parametric tests were used. The reproducibility of baseline values (pre-stretching measurements) between the first and second visits was assessed using the intraclass correlation coefficient (ICC) with 95% confidence intervals, typical error (TE), and coefficient of variation (CV), where appropriate. ICC estimates and 95% confidence intervals were calculated using a two-way mixed-effects model, an absolute-agreement definition, and single measurements [29]. This specification was selected because the same standardized procedures were applied at both visits and the objective was to quantify agreement in single-session values and pre-to-post change scores across repeated occasions. In addition, differences in baseline values between visits were examined using a paired-samples *t*-test. To assess the acute effect of static stretching and its comparability between visits, a two-way repeated-measures analysis of variance (ANOVA) was used, with time (pre- and post-stretching) and visit (first and second visit) as within-subject factors. The main effect of time, the main effect of visit, and their interaction (time × visit) were analyzed. Effect sizes were reported using partial eta squared (η^2^p). For additional comparison of post-intervention values between visits, a paired-samples *t*-test was also performed.

Reproducibility of the observed acute stretching response was assessed using individual pre-to-post change scores (post minus pre) at the first and second visits. ICC with 95% confidence intervals, TE, and CV were calculated for these differences. Bland–Altman analysis was additionally used to assess agreement between the acute responses observed at the two visits. For each participant, the difference between the responses at Visit 1 and Visit 2 was plotted against their mean. Systematic bias was calculated as the mean difference between visits (Visit 1 − Visit 2), and 95% limits of agreement (LoA) were calculated as the mean difference ± 1.96 × SD of the differences. Statistical significance was set at α = 0.05. ICC values were interpreted as indicating poor (<0.50), moderate (0.50–0.75), good (0.75–0.90), and excellent (>0.90) reproducibility [29]. ICC point estimates were interpreted alongside their 95% confidence intervals to reflect uncertainty in the reliability estimates. CV was reported where mathematically appropriate and was interpreted descriptively in the context of the outcome; no universal threshold for acceptable absolute reliability was applied.

## 3. Results

### 3.1. Reproducibility of Baseline Measurements

The analysis of baseline reproducibility (pre-stretching measurements obtained during the first and second visits) demonstrated very high relative reliability for both the SRT and ADF ROM, as indicated by high ICC values (0.98 and 0.95, respectively) with narrow 95% confidence intervals. TE was low for both tests, indicating good stability of baseline measurements. CV was calculated only for ADF ROM and amounted to 4.28% (95% CI: 3.36–5.91%), describing baseline variation relative to the mean ADF ROM. For the SRT, CV was not considered an appropriate indicator because the mean values were close to zero while the standard deviations were relatively large, which would have resulted in a methodologically inappropriate and potentially misleading interpretation of CV. Overall, baseline measurements showed excellent relative reliability between visits (Table 1); reproducibility of individual pre-to-post change scores was evaluated separately.

Additionally, paired-samples *t*-tests were conducted to examine differences in baseline measurements (pre-stretching) between the first and second visits. No statistically significant differences were observed for either the SRT or ADF ROM. For the SRT, the difference between visits was not statistically significant (t(25) = −1.05, *p* = 0.306). Similarly, no statistically significant difference was found for ADF ROM (t(25) = −1.95, *p* = 0.062), although a trend toward higher values during the second visit was observed. These tests provided no evidence of systematic differences in baseline means between visits.

### 3.2. Interaction Between Visit and Time

The results of the two-way repeated-measures ANOVA for the SRT revealed a statistically significant main effect of time, indicating that static stretching resulted in a significant improvement in SRT performance (F(1,25) = 116.75, *p* < 0.001, η^2^p = 0.824). The main effect of visit was not statistically significant (F(1,25) = 0.65, *p* = 0.428, η^2^p = 0.025), nor was the interaction between time and visit (F(1,25) = 0.73, *p* = 0.401, η^2^p = 0.028). There was no evidence of a systematic difference in the mean acute SRT response between visits. Mean SRT performance increased from 3.30 ± 1.81 cm to 6.98 ± 1.64 cm (combined mean across both visits).

For ADF ROM, the two-way repeated-measures ANOVA revealed a statistically significant main effect of time, indicating that short-term static stretching resulted in a significant acute increase in range of motion (F(1,25) = 20.73, *p* < 0.001, η^2^p = 0.453). The main effect of visit did not reach statistical significance (F(1,25) = 3.11, *p* = 0.090, η^2^p = 0.110), nor was there a statistically significant interaction between time and visit (F(1,25) = 0.59, *p* = 0.450, η^2^p = 0.023). There was no evidence of a systematic difference in the mean acute ADF ROM response between visits. Mean range of motion increased from 29.31 ± 1.05° to 30.46 ± 1.00° (combined mean across both visits). Additionally, paired-samples *t*-tests were conducted to examine differences in post-stretching values between the first and second visits. Consistent with the ANOVA findings, no statistically significant differences in mean post-stretching values were observed for the SRT (t(25) = −0.47, *p* = 0.642) or for ADF ROM (t(25) = −1.32, *p* = 0.198). The absence of statistically significant differences in mean values does not establish agreement of individual change scores; this was evaluated separately using ICC, TE, and Bland–Altman analyses.

### 3.3. Reproducibility of Individual Pre-to-Post Change Scores

**Figure 4:**
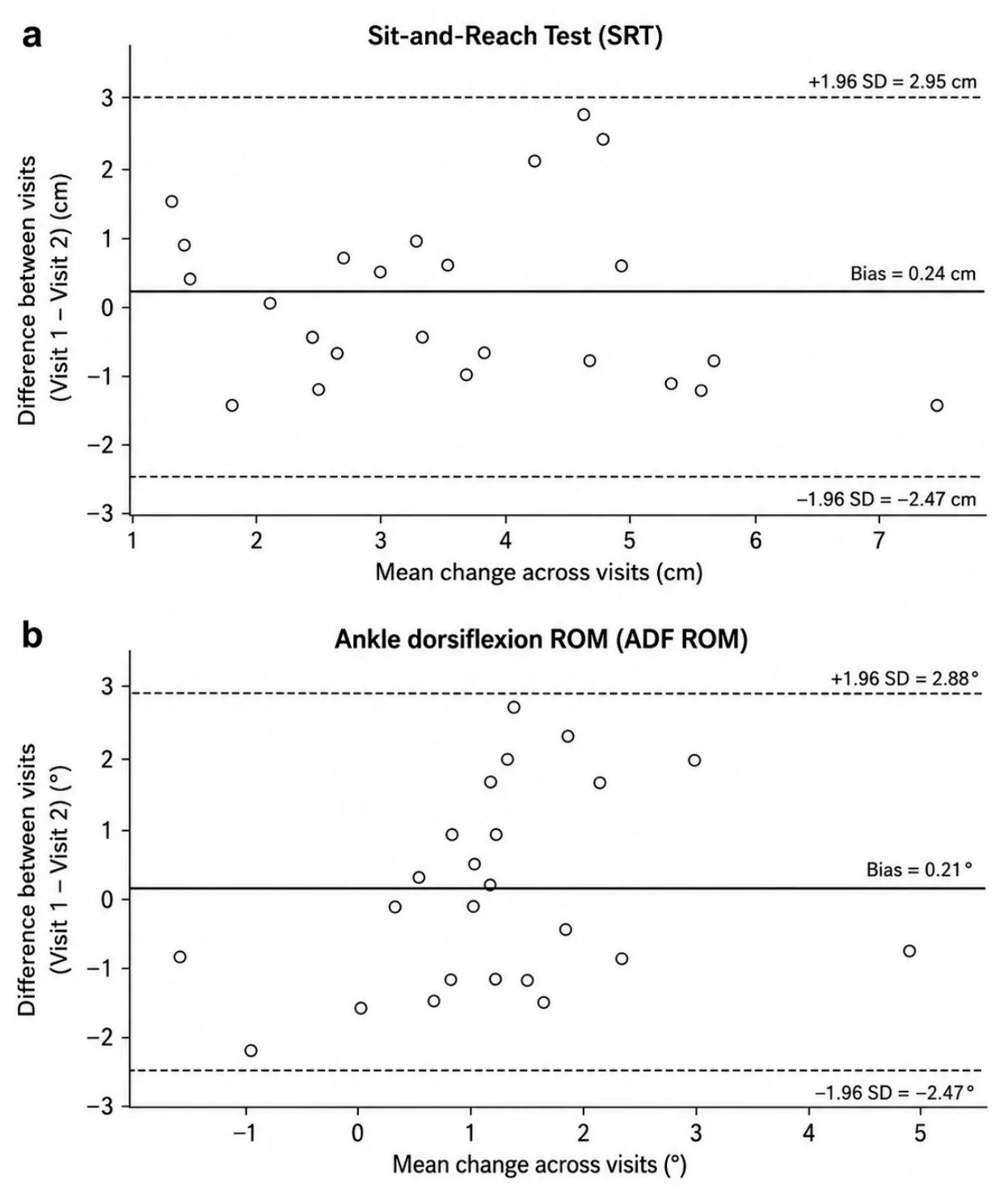
Bland–Altman plots showing agreement in individual pre-to-post change scores between Visit 1 and Visit 2 for (**a**) the Sit-and-Reach Test (SRT) and (**b**) ankle dorsiflexion range of motion (ADF ROM). The solid line represents the mean difference (bias), and the dashed lines represent the 95% limits of agreement (mean difference ± 1.96 SD).

Reproducibility of individual pre-to-post change scores in SRT performance and ADF ROM was assessed across the two visits. For the acute response, the SRT ICC was 0.74 (95% CI: 0.50–0.88), whereas the ADF ROM ICC was 0.57 (95% CI: 0.25–0.78); the latter interval spans values from poor to good reproducibility and therefore indicates substantial uncertainty around the point estimate (Table 2). The typical error of the repeated change scores, expressed as TE, was 0.98 cm for the SRT and 0.96° for ADF ROM. CV was considered appropriate only for the SRT and amounted to 26.57% (95% CI: 20.84–36.67%), indicating substantial variation in repeated individual change scores relative to the mean SRT change. For ADF ROM, CV was not reported because the mean changes were small and close to zero (with some participants even exhibiting reduced range of motion following the intervention), which would have resulted in a methodologically inappropriate interpretation of this metric. Bland–Altman analysis indicated little systematic bias between visits for either outcome. For the SRT, the mean difference between responses was 0.24 cm, with 95% LoA ranging from −2.47 to 2.95 cm. For ADF ROM, the mean difference was 0.21°, with 95% LoA ranging from −2.47° to 2.88° (Figure 4). Thus, although systematic differences between visits were small, the relatively wide limits of agreement indicate limited agreement of observed individual change scores across repeated interventions.

## 4. Discussion

In the present study, we investigated whether acute changes in range of motion following short-term static stretching of the hamstrings and gastrocnemius are reproducible. Individual pre-to-post change scores showed moderate reproducibility by point estimate, with considerable uncertainty (SRT: ICC = 0.74, 95% CI: 0.50–0.88; ADF ROM: ICC = 0.57, 95% CI: 0.25–0.78). SRT performance and ADF ROM increased significantly following stretching, and the mean pre-to-post response did not differ significantly between visits. Agreement of individual change scores across the two visits was limited, as indicated by the ICC, TE, and Bland–Altman analyses. Baseline measurements obtained prior to stretching demonstrated excellent reproducibility between visits for both outcomes.

The present design quantifies the reproducibility of observed pre-to-post change scores across repeated stretching sessions, but it cannot partition their variability into measurement error, normal day-to-day biological variation, and variability specifically attributable to the physiological response to stretching. Accordingly, the findings should be interpreted as evidence of limited reproducibility of individual change scores rather than as direct evidence of true biological intraindividual variability.

The excellent reproducibility of baseline measurements between visits (ICC = 0.98 for the SRT and ICC = 0.95 for ADF ROM) supports the stability of the measurement procedures. However, excellent baseline reliability does not establish reliable individual change scores or eliminate measurement error from those scores. Our findings regarding the reproducibility of the SRT and ADF ROM between visits are consistent with previous literature, as both individual studies and systematic reviews have reported excellent or good reproducibility of these tests [28,30,31,32].

Both SRT performance and ADF ROM increased significantly following stretching. This finding is consistent with a large body of evidence from both individual studies and meta-analyses demonstrating that short-term static stretching results in an acute increase in range of motion [22,23,24]. On average, SRT performance increased by 3.68 cm and ADF ROM by 1.15°, with large effect sizes observed for both tests. For ADF ROM, the TE of the repeated change scores (0.96°) was large relative to the mean change (1.15°). For SRT performance, the TE (0.98 cm) was smaller relative to the mean change (3.68 cm).

The lower ICC point estimate observed for ADF ROM should not be interpreted as evidence that the physiological response of the gastrocnemius is intrinsically less reproducible than the response reflected by SRT performance. The two stretching procedures differed in the degree of external control: hamstring stretching was passively imposed by the examiner, whereas gastrocnemius stretching was actively controlled by the participant. Because both procedures were progressed according to a subjective pain-threshold criterion, the actual stretching intensity may have varied more across sessions during the self-administered gastrocnemius stretch. Together with the small mean ADF ROM change relative to its TE, these methodological factors may partly account for the lower ICC point estimate for ADF ROM. The overlapping confidence intervals also preclude a firm conclusion that reproducibility differs between the two outcomes.

The mean response to stretching did not differ significantly between visits. Bryant et al. [26] similarly reported stable mean hamstring-stretching responses across five visits. However, similar mean responses do not establish agreement of individual pre-to-post change scores. Limited reproducibility of individual change scores has also been reported in studies of cardiorespiratory fitness and muscular adaptations to exercise training [17,18,20], despite similar mean responses to repeated interventions. For example, Del Giudice et al. [17] reported poor reproducibility of the response to two identical four-week high-intensity interval training interventions separated by a three-month washout period, both for VO2max (ICC = 0.369; CV = 74.4) and time to exhaustion (ICC = 0.048; CV = 45.6). Similarly, Islam et al. [18] found poor reproducibility of muscular adaptations, including protein content, enzymatic activity, and capillarization, following two identical high-intensity interval training interventions (ICC range: −0.42 to 0.04; CV range: 11–67).

The disagreement between repeated individual change scores may have several sources, which the present study cannot distinguish. A review of the literature revealed that most studies have focused on factors contributing to day-to-day fluctuations in baseline range of motion, whereas relatively few have addressed why the response to stretching itself may vary within an individual (i.e., why the increase in range of motion following a repeated application of the same intervention may be smaller or larger than during the initial intervention). Nevertheless, based on the available evidence and the physiological responses to stretching, several hypotheses for future testing can be proposed. Factors such as fatigue, sleep quality, stress, illness, and psychological state may influence observed changes after stretching [13]. These factors may, among other effects, influence motivation (i.e., the extent to which an individual exerts effort during testing) and pain tolerance [33,34]. For example, following a night of poor sleep, an individual may perceive pain sooner and therefore terminate the movement at an earlier point. Other potential contributors include ambient temperature [35], muscle damage (e.g., the presence of delayed-onset muscle soreness) [36], hormonal influences [37], hydration status [38], and muscle temperature or the degree of warm-up prior to testing [39,40]. Most of these factors are likely related to alterations in musculotendinous stiffness and/or stretch tolerance. This is plausible given that the acute increase in range of motion following a single bout of static stretching is thought to occur primarily as a result of reduced musculotendinous stiffness and increased stretch tolerance [24,25]. It is therefore possible that the extent to which these mechanisms change in response to stretching varies within an individual across different occasions. These mechanisms were not measured in the present study and should not be inferred from the observed change-score disagreement. Additional measurements, such as direct assessments of muscle stiffness, together with repeated control assessments, would be required to investigate their contribution.

The present study found mean improvements in SRT performance and ADF ROM following short-term static stretching. However, a single observed change score does not establish whether an individual has a reproducible intervention-specific response. Similar mean responses across visits provide little information about the agreement of a given participant’s change scores on repeated occasions. A single intervention protocol may therefore lead to misclassification of individuals. For example, a participant classified as a “non-responder” based on a single intervention could exhibit a positive response when the identical intervention is repeated, and vice versa. Consequently, we recommend that post hoc classifications of participants as “responders” and “non-responders” within intervention groups should be interpreted with considerable caution.

It is also worth noting that training outcomes are strongly influenced by behavioural and environmental factors, including adherence to the prescribed programme, psychosocial influences, nutrition, and testing conditions (e.g., time of day) [13]. This may help explain why exercise responses often appear highly variable in real-world settings, whereas findings from controlled and standardized research environments frequently provide limited statistical evidence for true interindividual variability in responsiveness. Variability observed in practice may therefore also reflect differences in how an intervention is implemented and adhered to. The present design cannot quantify these contributions or isolate differences in physiological responsiveness.

### Limitations

This study has several limitations. First, the sample size was relatively small (*n* = 26), and no a priori calculation targeting the precision of the reliability/reproducibility estimates was performed. The wide 95% confidence intervals, particularly for ADF ROM, indicate limited precision of the response ICC estimates. The sample size also limits the precision with which the Bland–Altman limits of agreement can be estimated. These findings should therefore be considered preliminary and confirmed in larger and more diverse samples. Only healthy young adults were included. Therefore, it remains unclear whether similar findings would be observed in older populations or in individuals with various pathologies. The sample was not stratified according to participation in sports requiring large ranges of motion or extensive flexibility-training experience, and the present study cannot determine whether response reproducibility differs in such populations. Another limitation is the absence of a control condition. Consequently, measurement error, normal day-to-day biological variation, and variability specifically attributable to the stretching response cannot be separated. Furthermore, the inclusion of a control group would have allowed us to assess whether, and to what extent, the baseline range-of-motion assessments themselves contributed to the observed increase in range of motion, as previous research has shown that testing alone can acutely increase range of motion, even when the assessment lasts only a few seconds [41]. It is also important to acknowledge that pain tolerance varies between individuals and may also vary within the same individual across different occasions [33,34]. Consequently, stretching intensity may not have been identical between participants or within participants across the two visits, as stretching was performed to the pain threshold and range-of-motion assessments were performed to the end of the available range (participants were instructed to stretch “as far as possible, into pain”). In addition, participants may have interpreted the concepts of “pain threshold” and “stretching as far as possible” differently. Although this subjectivity represents a limitation of the present study, it should be noted that it is inherent to most research on flexibility, as it is difficult to establish an objective and standardized stretching intensity [25]. Finally, the present study identified limited reproducibility of observed change scores but did not determine the mechanisms underlying their disagreement. Future studies should combine repeated intervention and control assessments with measures of potential physiological and behavioural contributors.

No separate familiarization session or additional baseline-only visit was conducted. The seven-day interval was selected to minimize carryover from the acute stretching session. Baseline values did not differ significantly between visits and demonstrated excellent relative reliability; nevertheless, residual carryover, adaptation, or familiarization effects cannot be completely excluded.

## 5. Conclusions

Short-term static stretching was followed by similar mean increases in SRT performance and ADF ROM across the two visits, but individual pre-to-post change scores showed only moderate and imprecisely estimated reproducibility (SRT: ICC = 0.74, 95% CI: 0.50–0.88; ADF ROM: ICC = 0.57, 95% CI: 0.25–0.78). The present design cannot determine how much of the disagreement between repeated change scores reflects measurement error, normal day-to-day variation, or variability specifically attributable to the stretching response. Accordingly, classifications of individuals as “responders” or “non-responders” based on a single acute stretching session should be interpreted cautiously. Given the modest sample size, these findings should be confirmed in larger and more diverse samples, preferably with repeated control assessments.

## Figures and Tables

**Table 1:** Reproducibility of baseline measurements (pre-stretching) between the first and second visits.

Variable	Visit 1	Visit 2	ICC	95% CI	TE	95% CI	CV	95% CI
SRT	3.09 ± 9.22	3.51 ± 9.37	0.98	0.95–0.99	1.46	1.14–2.01	–	–
ADF ROM	28.97 ± 5.36	29.65 ± 5.44	0.95	0.89–0.98	1.26	0.98–1.73	4.28	3.36–5.91

SRT—Sit-and-Reach Test, ADF ROM—ankle dorsiflexion range of motion test, ICC—intraclass correlation coefficient, CI—confidence interval, TE—typical error, CV—coefficient of variation. TE is expressed in the original units of each outcome (cm for SRT; degrees for ADF ROM).

**Table 2:** Reproducibility of Individual Pre-to-Post Changes in SRT Performance and ADF ROM Between the First and Second Visits.

Variable	Visit 1	Visit 2	ICC	95% CI	TE	95% CI	CV	95% CI
Change in the SRT	3.80 ± 1.61	3.57 ± 2.10	0.74	0.50–0.88	0.98	0.77–1.35	26.57	20.84–36.67
Change in ADF ROM	1.24 ± 1.55	1.04 ± 1.34	0.57	0.25–0.78	0.96	0.76–1.33	–	–

SRT—Sit-and-Reach Test; ADF ROM—ankle dorsiflexion range of motion test; ICC—intraclass correlation coefficient; CI—confidence interval; TE—typical error, CV—coefficient of variation. TE is expressed in the original units of each outcome (cm for SRT; degrees for ADF ROM).

## Data Availability

The data collected in this manuscript are available freely in the Zenodo database (DOI: 10.5281/zenodo.22029634/; URL: https://zenodo.org/records/22029635, accessed on 16 September 2026).

## Abbreviations

The following abbreviations are used in this manuscript:

ADF ROM	Ankle dorsiflexion range of motion
ANOVA	Analysis of variance
CI	Confidence interval
CV	Coefficient of variation
ICC	Intraclass correlation coefficient
SRT	Sit-and-Reach Test
TE	Typical error
